# Peer Support in Psychosis Care: A Valuable Resource for Recovery

**DOI:** 10.1192/j.eurpsy.2024.801

**Published:** 2024-08-27

**Authors:** F. Cunha, I. Santos, N. Castro, R. Andrade, E. Almeida, J. Abreu, J. Martins, R. Vaz, S. Borges

**Affiliations:** ^1^Centro Hospitalar Tondela-Viseu , Viseu, Portugal

## Abstract

**Introduction:**

A variety of peer support workers have been integrated in the mental health workforce in several countries. The effectiveness of this approach is still inconclusive. However, some data reveals promising results. Some projects have integrated peer support intervention in the treatment of psychosis. In fact, UK clinical guidelines for psychosis advise the inclusion of peer support within Early Intervention in Psychosis services.

**Objectives:**

The current study aims to evaluate how peer support may assist the intervention in psychosis and highlight challenges ahead in this field.

**Methods:**

Narrative review of the available scientific literature.

**Results:**

Research suggests that consistent and frequent peer support enhances social support and boosts self-confidence and the overall quality of life for people going through psychosis. Individuals diagnosed with severe mental illnesses who receive peer support reportedly experience an increased sense of control, hopefulness, and empowerment, enabling them to initiate positive changes in their lives. People going through psychosis experience internalized stigma. Destigmatization of psychotic experiences is a central theme of intervention in psychosis. Participants viewed peer support as a valuable form of assistance that could offer advantages to both peers (service users) and peer support workers.

**Conclusions:**

Peer support makes a strong contribution to destigmatising psychosis. The available date is promising and supports the effectiveness of peer support in such instances. As projects of peer support in psychosis continue to be implemented, further research should provide additional insight into the effectiveness and inherent challenges of this type of intervention.

**Disclosure of Interest:**

None Declared

